# RNA Interference of Chitin Synthase 2 Gene in *Liriomyza trifolii* through Immersion in Double-Stranded RNA

**DOI:** 10.3390/insects13090832

**Published:** 2022-09-13

**Authors:** Ya-Wen Chang, Yu-Cheng Wang, Yu-Qing Yan, Hong-Fang Xie, Deng-Rong Yuan, Yu-Zhou Du

**Affiliations:** 1 School of Horticulture and Plant Protection & Institute of Applied Entomology, Yangzhou University, Yangzhou 225009, China; 2 Plant Protection and Quarantine Station of Nanjing, Nanjing 210000, China; 3 Joint International Research Laboratory of Agriculture and Agri-Product Safety, The Ministry of Education, Yangzhou University, Yangzhou 225009, China

**Keywords:** *Liriomyza trifolii*, chitin synthase 2 (*CHS2*) gene, immersion of dsRNA, RNAi, emergence rate

## Abstract

**Simple Summary:**

*Liriomyza trifolii* is an important insect pest that infects many horticultural crops and vegetables, displaying strong interspecific competitiveness and inflicting serious harm. Here, chitin synthase 2 transcript was studied, and a prepupal immersion dsRNA delivery method was established for *L. trifolii*. The dsRNA can enter in the prepupal stage and play a role in the pupal stage, resulting in a decrease in the *CHS2* expression level and eclosion rate. This study is an important supplement to the research on the RNAi of *L. trifolii*, enhances knowledge of the function of chitin synthase 2 in *Liriomyza* species, and may also provide a new idea for its control strategies.

**Abstract:**

*Liriomyza trifolii* is an important invasive pest that infects horticultural vegetables, displaying a strong competitive advantage and showing great potential for inflicting harm. Chitin synthase is one of the key enzymes in insect chitin metabolism and plays an important role in insect growth and development. In this study, a chitin synthase (CHS) transcript of *L. trifolii* was cloned, and the results showed that *LtCHS* belongs to the CHS2 family. The expression analysis indicated the presence of the highest abundance of *LtCHS2* in the pupae at different developmental stages but showed no significant difference among different tissues in the adult. Furthermore, a dsRNA immersion method was developed for RNA interference (RNAi) in *L. trifolii* using *LtCHS2* transcript. RNAi can significantly reduce the expression of *LtCHS2* in pupae, and the emergence rate of the pupae was significantly lower than that of the control. The results provide a theoretical basis for exploring the role of chitin synthase gene in *L. trifolii* and proposing new pest control strategies.

## 1. Introduction

*Liriomyza trifolii* (Diptera: Agromyzidae) is a pest infecting horticultural crops across the world and an invasive insect pest in both field and greenhouse settings in China [1,2]. The larvae of *L. trifolii* damage plants by producing tunnels in leaf tissue, whereas the adults pierce the foliage during oviposition [3]. In recent years, with the development of facility agriculture, the damage inflicted by *Liriomyza* spp. is becoming increasingly serious. In China, *L. trifolii* is mainly distributed in the southeast coastal areas, while recent investigations show that *L. trifolii* has invaded the northern regions of China, reaching as far as Hengshui city (37–38° N) [4,5]. Although pesticides have been an effective and convenient option for controlling *L. trifolii*, their irrational use has created resistance among pests. Together with other competitive factors, pesticide resistance will change the current population pattern of *Liriomyza*, increasing the threat of *L. trifolii*. In general, *L. trifolii* has obvious competitive advantages compared with species closely related to it [6,7,8].

RNA interference (RNAi) is a highly specific approach that suppresses gene expression at the post-transcriptional level. It can be used to investigate gene function [9]. In addition, ingested double-stranded RNA (dsRNA) may also have insecticidal properties [10,11]. However, in the RNAi-based crop protection method, the delivery of dsRNA is a major challenge. After identifying the target gene, it is important to choose a convenient strategy to deliver dsRNA to insects. Microinjection is a good strategy for functional genomic studies, but this method is not suitable for controlling insect pests in the field. Furthermore, microinjection has some limitations. For example, it is highly technical and difficult to achieve in some tiny insects [12]. Feeding methods require insects to feed and achieve RNAi goals through midgut absorption. However, midgut barriers lead to the low efficiency of RNAi in some insects [12]. It is feasible to supply dsRNA to herbivorous insects through root absorption or injection into plant tissues [13,14], and the direct immersion method is also carried out in nematode and corn borers [15,16]. Furthermore, plant-mediated RNAi and nanomaterial-mediated RNAi delivery is also being carried out, improving RNAi efficiency [12,17].

Insect chitin synthase (CHS) is one of the key enzymes in the process of insect chitin metabolism. It plays an important role in the growth and development of insects. It is also an ideal potential target gene for the development of new insecticides and has a high research value [18]. The chitin synthase gene was first obtained from *Lucilia cuprin* in 2000 [19], after which the chitin synthase sequences of other insects, such as *Drosophila melanogaster*, *Tribolium castaneum*, and *Spodoptera frugiperda*, were also cloned [20,21,22]. Chitin synthase gene sequences of different species were analyzed. It was found that chitin synthase genes can be divided into CHS1 and CHS2 [23]. CHS1 is mainly expressed in the outer embryonic layer cells, which is responsible for the synthesis of chitin in epidermis and trachea, while Chs2 is mainly expressed in the epithelial cells of midgut peritrophic membrane, which is responsible for the synthesis of chitin in midgut peritrophic membrane [18,19]. Many studies have used RNAi to inhibit the expression of CHS. The injection of small interfering RNA (siRNA) targeting *CHS1* to knock down *CHS1* transcripts at larval, pupal, and adult stages of *Culex pipiens pallens* has resulted in the appearance of different lethal phenotypes. When larval and pupal stages were injected with *siCHS1*, *CHS1* knockdown prevented the growth and development of different insect stages and impaired the production of chitin and chitin degradation, which resulted in an ecdysis defect phenotype of mosquitoes [24]. In *Henosepilachna vigintioctopunctata*, *dsCHS1* and *dsCHS2* were used to immerse potato foliage and the treated leaves were provided to the newly molted larvae. The knockdown of *HvCHS1*, rather than *HvCHS2*, affected its growth and metamorphosis [25]. In *Cnaphalocrocis medinalis*, *CmCHS2* was expressed throughout development and in all of the adult tissues tested, with the highest expression level in the adult and in the midgut. Silencing of *CmCHS2* severely affected *C. medinalis* larval growth and caused larval lethality [26].

In this study, *CHS2* transcript, the key to the growth and development of *L. trifolii*, was examined. Prepupal immersion in dsRNA was used to explore its function in the growth and development of *L. trifolii*. This research is of great significance to exploring the role of *CHS2* in *L. trifolii* and developing a new control strategy based on RNAi.

## 2. Materials and Methods

### 2.1. Insects

*L. trifolii* populations were collected from Yangzhou (32.39° N, 119.42° E), China, and reared in the laboratory on kidney bean plants for more than five years at 26 °C with a 16:8 h (L:D) photoperiod as described [27]. Kidney bean plants were used to feed larvae and adults, and foliage with tunnels was collected for pupation.

### 2.2. Cloning, Sequence Alignment, and Expression of CHS2 Transcript

The RNeasy reagent (Vazyme, Nanjing, China) was used to isolate total RNA from *L. trifolii* pupae. RNA quality, purity and integrity were determined by spectrophotometry (Thermo NanoDrop One, Madison, WI, USA) and agarose gel electrophoresis. On the basis of previously published transcriptome data [28], we selected chitin synthase 2 transcript for further study. A partial fragment of the chitin synthase 2 transcript was amplified using specific primers (Table 1), and 5′- and 3′-Rapid amplification of cDNA ends (5′- and 3′-RACE) was used to obtain complete cDNAs as described [29].

Full-length cDNA of the chitin synthase 2 (CHS2) was queried against other Diptera chitin synthase genes using BLAST programs (http://www.ncbi.nlm.gov/BLAST/, accessed on 10 May 2022). Clustal X was used to align sequences [30], and open reading frames (ORFs) were identified with ORF Finder (https://www.ncbi.nlm.nih.gov/orffinder/, accessed on 10 May 2022). Tools available on the ExPASy Molecular Biology Server (https://prosite.expasy.org/, accessed on 10 May 2022) were deployed to scan specific motifs in the chitin synthase gene. MEGA software [31] and the neighbor-joining method were used to create phylogenetic trees of chitin synthase genes.

To explore the function of *LtCHS2*, qRT-PCR was used to study the expression pattern of *LtCHS2* in different developmental stages and tissues. The developmental stages included 3rd instar larvae, prepupa, two-day-old pupae (new pupae), seven-day-old pupae (old pupae), female adults, and male adults (*n* = 10). The different adult tissues used were from head, thorax, abdomen, and gut, and the treatments contained three independent biological replicates. Total RNA (0.5 μg) was reverse-transcribed using the HiScript II Q RT SuperMix for qPCR (+gDNA wiper) (Vazyme, Nanjing, China). qRT-PCR was executed with gene-specific primers (Table 1) in 20 µL volumes, comprised 10 μL ChamQ SYBR qPCR Master Mix (2×) (Vazyme, Nanjing, China), 1 μL of each gene-specific primer (10 μM) (Table 1), 2 μL of cDNA template, and 6 μL of ddH_2_O as described [32]. Reactions were conducted with a CFX-96 real-time PCR system (Bio-Rad Laboratories, Berkeley, CA, USA) under the following conditions: 3 min at 95 °C, 39 cycles of denaturation at 95 °C for 30 s, and annealing at the T_m_ of primer pairs (Table 1) for 30 s. Each treatment contained four replicates, and each reaction was performed in triplicate.

### 2.3. Synthesis and Delivery of dsRNA

Full-length *L. trifolii* chitin synthase cDNA sequence was analyzed with siDirect v. 2.0 (http://sidirect2. rnai.jp/, accessed on 10 May 2022) to select potential small interfering RNA (siRNA) sequences that could be used to design dsRNA primers. Forward and reverse primers included a T7 promoter sequence (TAATACGACTCACTATAGGGAGA) at the 5′ ends to catalyze transcription from both cDNA strands (Table 1). As a control, dsRNA specific to the gene encoding green florescent protein (*GFP*) was used (Table 1). PCR products were inserted into pGEM-T easy vector (Promega, Madison, WI, USA), and resulting constructs were used as template DNA in subsequent amplifications. Purified DNA templates (1.5 µg) were used for in vitro dsRNA synthesis and purified using the MEGAscriptTM RNAi Kit (Thermo, Waltham, MA, USA) according to manufacturer’s protocol. The quality and integrity of dsRNA was evaluated by spectrophotometry and gel electrophoresis.

We selected the prepupa that had just left the leaf as the experimental insect developmental stages, placed them into the Petri dish, and used different concentrations of dsRNA and 1% RNATransMate (Sangon Biotech, Shanghai, China) to immerse the prepupa. After 10 s immersion, we removed the excess droplets of dsRNA using a soft brush, to prevent it from blocking the stomata and affecting pupation, and used the prepupa for subsequent experiments. Each treatment involved 10 prepupae, each treatment was repeated 3 times, and *dsGFP* was used as the control.

### 2.4. Analysis of Silencing Efficiency

Concentration experiments were performed using the experimental design described above to evaluate the impact of dsRNA delivery on the pupae. The experiment was performed with three replicates per treatment, and *dsGFP* was used as a control. Three concentrations (dsCHS: 300, 600, and 900 ng/μL; dsGFP: 600 ng/μL) were selected for further analysis, and silencing efficiency was determined 2 days after pupation. Each treatment involved 30 pupae (*n* = 30). The pupae were collected for RNA extraction, and silencing efficiency was analyzed by qRT-PCR. In addition, survival rates were calculated for the 600 ng/μL dsRNA group, containing 30 pupae (10 individuals representing one repetition). The number of eclosion adults was recorded.

### 2.5. Statistical analysis

Expression levels of *CHS2* were identified using the 2^−ΔΔCt^ method [33], and *Actin* and *18S* served as reference genes [32]. Relative transcript abundance was calculated using the average Cq values of the two reference genes. The expression of the *CHS2* transcript under different developmental stages, using tissues and different dsRNA concentrations, was analyzed with one-way ANOVA, followed by Tukey’s multiple comparison and analysis with SPSS v. 16.0. For ANOVA, data were tested for homogeneity of variances using Levene’s test and transformed to follow a normal distribution. In addition, Student’s t-test was used to compare differences in mortality with SPSS v. 16.0, and differences were considered significant at *p* < 0.05.

## 3. Results

### 3.1. Sequence Characteristics and Phylogenetic Analysis of CHS2 Transcript from L. trifolii

**Figure 1:**
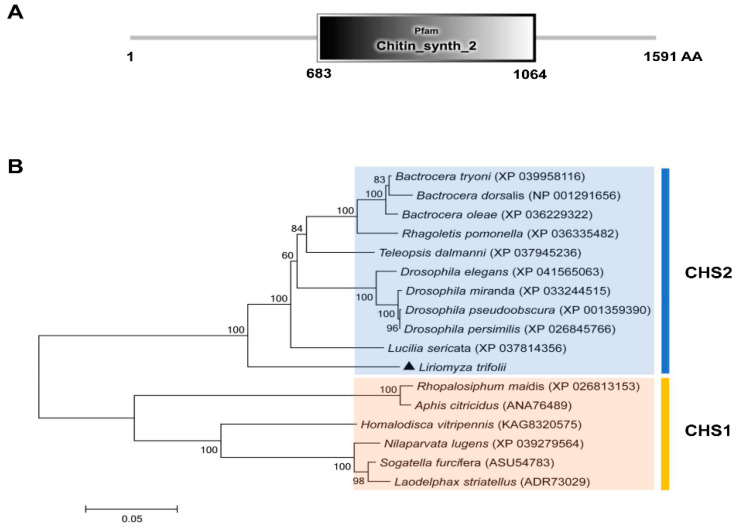
The domain organization of CHS2 (**A**) and the neighbor-joining phylogenetic tree of CHS2 (**B**). *L. trifolii* CHS2 is labeled with triangles. Numbers on the branches represent bootstrap values obtained from 1000 replicates (only bootstrap values >50 are shown).

In this study, a *CHS2* cDNA sequence was cloned in *L. trifolii*. The full length is 5650 bp and the open reading frame (ORF) is 4776 bp long. It is predicted to encode 1591 amino acids, has a molecular weight of 180.41 kDa and an isoelectric point of 6.38. The sequence was submitted to GenBank as accession no. ON453844. The characteristic sequence of the CHS2 gene family was found to be located in the amino acid sequence encoded by the CHS2 gene (chitin_synth_2: 683-1064) (Figure 1A and Figure S1). In addition, the characteristic sequences of chitin synthase (EDR and QRRRW) were also found (Figure S1). A phylogenetic tree was obtained using the amino acid sequences of 16 chitin synthases in Diptera, including 10 chitin synthase 2 and 6 chitin synthase 1 sequences. The phylogenetic tree contained two distinct clusters containing chitin synthase 1 and chitin synthase 2. The results showed that CHS2 of *L. trifolii* formed a branch of chitin synthase 2 and was close to *Lucillia sericata* (Figure 1B).

### 3.2. Expression Pattern of CHS2 Transcript in L. trifolii

**Figure 2:**
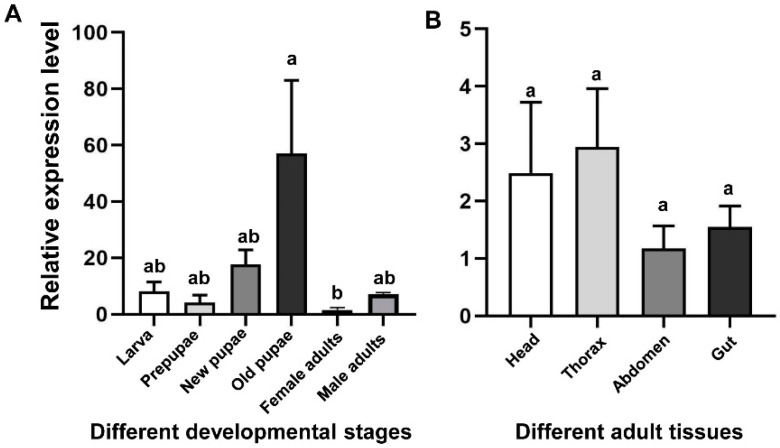
Transcript relative expression analysis of *CHS2* in *L. trifolii* at different developmental stages (**A**) and adult tissues (**B**). Different lowercase letters in panels indicate significant differences between treatments. Tukey’s multiple range test was used for pairwise comparison of means (*p* < 0.05).

qRT-PCR was used to detect the mRNA levels of *CHS2* transcript in different developmental stages (larvae, prepupae, new pupae, old pupae, and female and male adults) and in different tissues from the adult (head, thorax, abdomen, and gut) (Figure 2). The results showed that the expression levels of *CHS2* transcript in *L. trifolii* were significantly different at different developmental stages. The expression level in the old pupae was the highest, 16.17-fold higher than that in the control group (female adults) (*F*_5,12_ = 4.230; *p* < 0.05) (Figure 2A). However, in different tissues, there was no significant difference among adult tissues (*F*_3,8_ = 0.945; *p* = 0.463) (Figure 2B).

### 3.3. Functional Verification of CHS2 Transcript of L. trifolii

**Figure 3:**
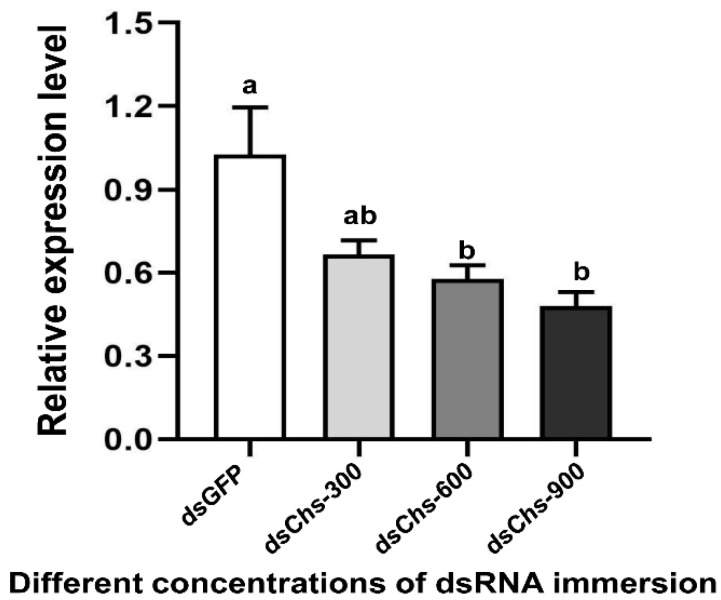
Relative expression levels of *CHS2* under immersion treatments with different concentrations of dsRNA. The data are denoted as the mean ± the SE. One-way analysis of variance (ANOVA) was used to analyze the relative expression levels of CHS2 under different treatments. Different lowercase letters indicate significant differences among different temperature treatments. Tukey’s multiple range test was used for pairwise comparison for mean separation (*p* < 0.05).

**Figure 4:**
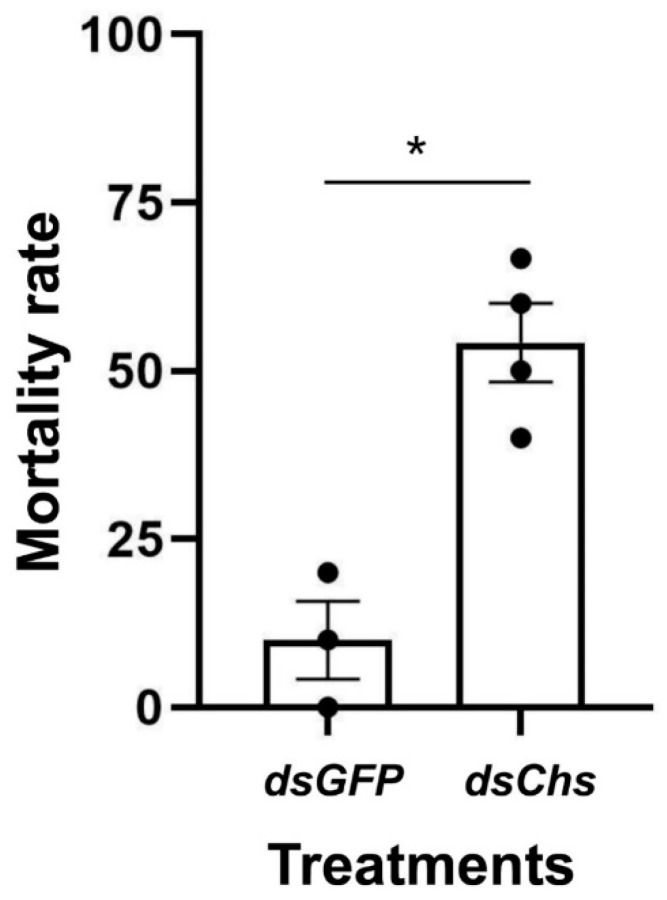
RNAi-mediated knockdown of *CHS2* decreases *L. trifolii* survival. Percentage of mortality in insects immersed in *dsCHS2* and *dsGFP* (control). The data are denoted as the mean ± the SE. Dots represent individual repetitions. Data were analyzed by Student’s *t*-test, *p* < 0.05. Asterisks represent significant differences in mortality data.

RNA interference studies were conducted by immersing *L. trifolii* prepupae in the dsRNA of *CHS2*. There was a significant difference in the *CHS2* expression levels in *L. trifolii* with 600 and 900 ng/μL of *dsCHS2*, which were 56.86 and 56.04% of the *dsGFP* control group, respectively (*F*_3,8_ = 8.207; *p* < 0.05). However, *CHS2* expression was not significantly different with 300 ng/μL of *dsCHS2* compared with the *dsGFP* control group (Figure 3). In terms of mortality, compared with the *dsGFP* control group, the mortality rate of *L. trifolii*, when immersed in *dsCHS2* at the prepupal stage, was significantly higher than that of the *dsGFP* control group, which were 54.17% and 10%, respectively (*t* = 5.242; *p* < 0.05) (Figure 4).

## 4. Discussion

Chitin synthase (CHS) plays a critical role in the synthesis of insect cuticle [18,34]. However, the current research on *CHS* is still focused on some model insects, and there is far more research on *CHS1* than on *CHS2* in terms of gene types [20,21,24,25]. Therefore, the experimental object of this study was *L. trifolii*, and the *CHS2* cDNA sequence and the function of *CHS2* in *L. trifolii* were extensively studied. The *CHS2* transcript of *L. trifolii* was originally obtained from previous transcriptome data of *L. trifolii* [28] and full-length cloning and sequencing in this study. CHS contains characteristic sequences of the CHS2 gene family, including the characteristic sequences EDR and QRRRW unique to chitin synthase, which exist in all types of chitin synthase [19]. CHS2 obtained in this study is distributed in the CHS2 family region in the phylogenetic tree, which lays a solid foundation for further study of this gene in *L. trifolii*.

Chitin synthase has different expression patterns in different developmental stages and tissues, so its functions are also different. The expression pattern results of *CHS2* of *L. trifolii* show that *CHS2* transcript is expressed in each growth and development stage of *L. trifolii*, indicating that the *CHS2* gene plays a role in each development stage of *L. trifolii*. However, the expression levels vary in different development stages. The expression levels of *CHS2* were the highest in old pupae and lower in larvae and new pupae, but the expression levels at the prepupae and adult stages were the lowest. This expression pattern is different from the results of previous studies on *Grapholita molesta*. *GmCHS2* is less expressed in the larval stage, and the expression will suddenly increase in prepupae, while the expression in the pupal stage is low but the expression in the adult stage is significantly higher than that in the larval stage [35]. In addition, although the expression of *D. melanogaster* in the larval stage is relatively low, its expression increases significantly in prepupae [20]. This difference may be related to the feeding habits of different insect species and the habit of the *L. trifolii* larvae to feed inside leaves, which is significantly different from the habits of other insects. At the same time, *L. trifolii* needs to leave the leaf to pupate in the prepupal stage and changes in environmental conditions may also lead to changes in the expression of *CHS2*, but the mechanism is still unknown. The expression of *CHS2* increased significantly in old pupae. *L. trifolii* is about to emerge in this developmental stage, and the *CHS2* gene may be involved in the process of metamorphosis and development of *L. trifolii* and the imminent life activities, such as feeding and mating, after emergence, which is similar to the pattern of high expression of *CHS2* in the pupal stage in *Culex pipiens quinquefasciatus* and *Anopheles gambiae* [36]. In general, as mentioned above, the expression patterns of *CHS2* in different insects at different developmental stages are significantly different. Therefore, it is speculated that the development duration criteria of different insects may be different and may also be related to the specific living habits of different insects and other functions of the *CHS2* gene at certain developmental stages, which need to be further studied. In addition, in different tissues of *L. trifolii*, *CHS2* was expressed in the head, thorax, abdomen, and gut. The analysis of variance showed that there was no significant difference among different tissues. Relevant studies have shown that *CHS2* participates in the synthesis of the peritrophic membrane of gut epithelial tissue, so it is mainly expressed in the insect gut [37]. In this study, the expression of *CHS2* in the gut was also higher than that in other abdominal tissues (noted after intestinal dissection), although there was no significant difference in the results. The high expression of *CHS2* in other tissues suggests that *CHS2* may have other functions in different tissues.

RNA interference is a phenomenon of gene silencing at the post transcriptional level, and it has been widely used in the study of gene function [12]. The object of this study was *L. trifolii*, and the *CHS2* transcript function was studied extensively. RNAi technology in *L. trifolii* adults has been reported previously. However, due to the tiny size of *L. trifolii* adults, the mortality caused by microinjection is relatively high. The limitations of microinjection make exploring the function of genes specifically expressed in other insect states challenging [38]. The immersion method is applicable to the RNAi of some pests, and relevant research on RNAi mediated by nanomaterials may help improve the efficiency of RNAi [10,12,39]. In this study, RNAi delivery was carried out by the direct immersion method. Through the biological habit of *L. trifolii*, the larvae left the leaves to pupate and were immersed in the prepupal stage of the key period. The prepupal stage can turn into pupae within a few hours [40], and dsRNA can be incorporated into the body to achieve dsRNA delivery. In this experiment, after *dsCHS2* immersion in the prepupal stage, qPCR results showed that the *CHS2* was significantly reduced after interference and the mortality rate of the adults was significantly increased. In this study, we chose only *dsGFP* with a concentration of 600 ng/μL for immersion treatment, based on the fact that *dsGFP* is a control and its synthetic reagents are the same as the target dsRNA. Therefore, we have reason to believe that the concentration of *dsGFP* in this study does not affect the survival of *L. trifolii* and the expression of the target *LtCHS2*. However, it is worth noting that the length of dsRNA and concentration of the controls still need to be carefully considered in each RNAi experiment. In other studies, when the larvae of *T. castaneum* were injected with *dsTcCHS2*, the larvae shrunk due to hunger and the peritrophic membrane was basically no longer generated [21]. Injecting *dsCHS2* in the third instar silkworm larvae, most of which do not molt or do not molt normally, interferes with the expression of *BmCHS2* [41]. When the second and fourth instar potato beetle larvae were fed *dsCHS2*, the larvae ate less and grew slowly, and the content of chitin in the midgut decreased [42]. Feeding *dsCHS2* had no effect on the larval stage of *Spodoptera exigua* but had a certain effect on prepupae and adults, and 20% and 25% could not pupate and eclose, respectively [43]. Similarly, in a study on locusts, the intake of locusts injected with *dsCHS2* was significantly reduced, and the length of the midgut was significantly shorter [44]. In addition, in a study of *Anopheles gambiae*, chitosan-coated dsRNA was used to interfere with *AgCHS2*. The results showed that the content of chitin in its peritrophic membrane decreased, affecting its growth and development [45]. Therefore, chitin synthase 2 plays an important role in insect growth and development. However, in this study, the average life span is shorter and feeding is limited for *Liriomyza* adults, which makes some physiological observations impractical.

## 5. Conclusions

RNAi-based gene function research and pest control strategies have been tried in some species, but most of the current research is still in its infancy. In this study, *CHS2*, an important growth and development gene, was selected as the target gene and the prepupal immersion method was used to verify the function of the *CHS2* gene. In general, dsRNA can enter in the prepupal stage and play a role in the pupal stage, resulting in a decrease in the *CHS2* transcript expression level and eclosion rate. The results provide a theoretical basis for exploring the role of the chitin synthase gene in *L. trifolii* and may propose new pest control strategies.

## Figures and Tables

**Table 1:** Primers used in cDNA cloning, dsRNA synthesis, and real-time quantitative PCR.

**Gene**		**Primer Sequences (5′** **→3′)**	**Length** **(bp)**	**Tm** **(°C)**	
Primers for cDNA cloning and full-length cDNA amplification					
*CHS2*	F	TACACACTGAGGGTTCCCATTT	999	53.9	
	R	GAACTCATTGAAACCTTCTGGA			
	5′	TTGTCATCGCTCTCACCCGCTCC	393	64.5	
	3′	CTGCGAATCTGACAGGGCGTTTG	1566	61.6	
Primers for dsRNA synthesis					
*dsCHS2*	F	TAATACGACTCACTATAGGGTTAGTTTGGATTTTACCTGGCA	553	63.1	
	R	TAATACGACTCACTATAGGGTTCTACACGATAGCCTCTTTGC			
*dsGFP*	F	TAATACGACTCACTATAGGGCCTCGTGACCACCCTGACCTAC	314	68.2	
	R	TAATACGACTCACTATAGGGCACCTTGATGCCGTTCTTCTGC			
**Primers for qRT-PCR**			**Length** **(bp)**	**Tm** **(°C)**	**Efficiency (%)**
*qPCR-CHS2*	F	TACACACTGAGGGTTCCCATTTA	116	59.0	97.5
	R	TCCATCATTTCATCCTTAGTTTC			
*qPCR-Actin*	F	TTGTATTGGACTCTGGTGACGG	73	59.2	108.6
	R	GATAGCGTGAGGCAAAGCATAA			
*qPCR-18S*	F	GAAGCAGTTTGGGGGCATTA	88	60.0	103.2
	R	TTGGCAAATGCTTTCGCTTA			

Note: F, forward; R, reverse; 5′, 5′ RACE primer; 3′, 3′ RACE primer; underscored nucleotides indicate the T7 polymerase promoter sequence.

## Data Availability

Data is contained within the article and Supplementary Material.

## Supplementary Materials

The following are available online at https://www.mdpi.com/article/10.3390/insects13090832/s1, Figure S1: Nucleotide sequences for *L. trifolii* CHS2 cDNA and its predicted amino acid sequence. Nucleotide numbering starts with the adenine in the first methionine codon of the putative open reading frame. The highly conserved region, chitin_synth_2 domain, is highlighted. The asterisk indicates the translational termination codon. The EDR and QRRRW motifs are boxed.
