# Supplementary material for: RNA Interference of Chitin Synthase 2 Gene in Liriomyza trifolii through Immersion in Double-Stranded RNA
Source: Insects. 2022 Sep 13;13(9):832. doi: 10.3390/insects13090832 (PMC9504599; doi:10.3390/insects13090832)
Supplement: Supplementary file 1 [file insects-13-00832-s001.zip › Figure S1.pdf]

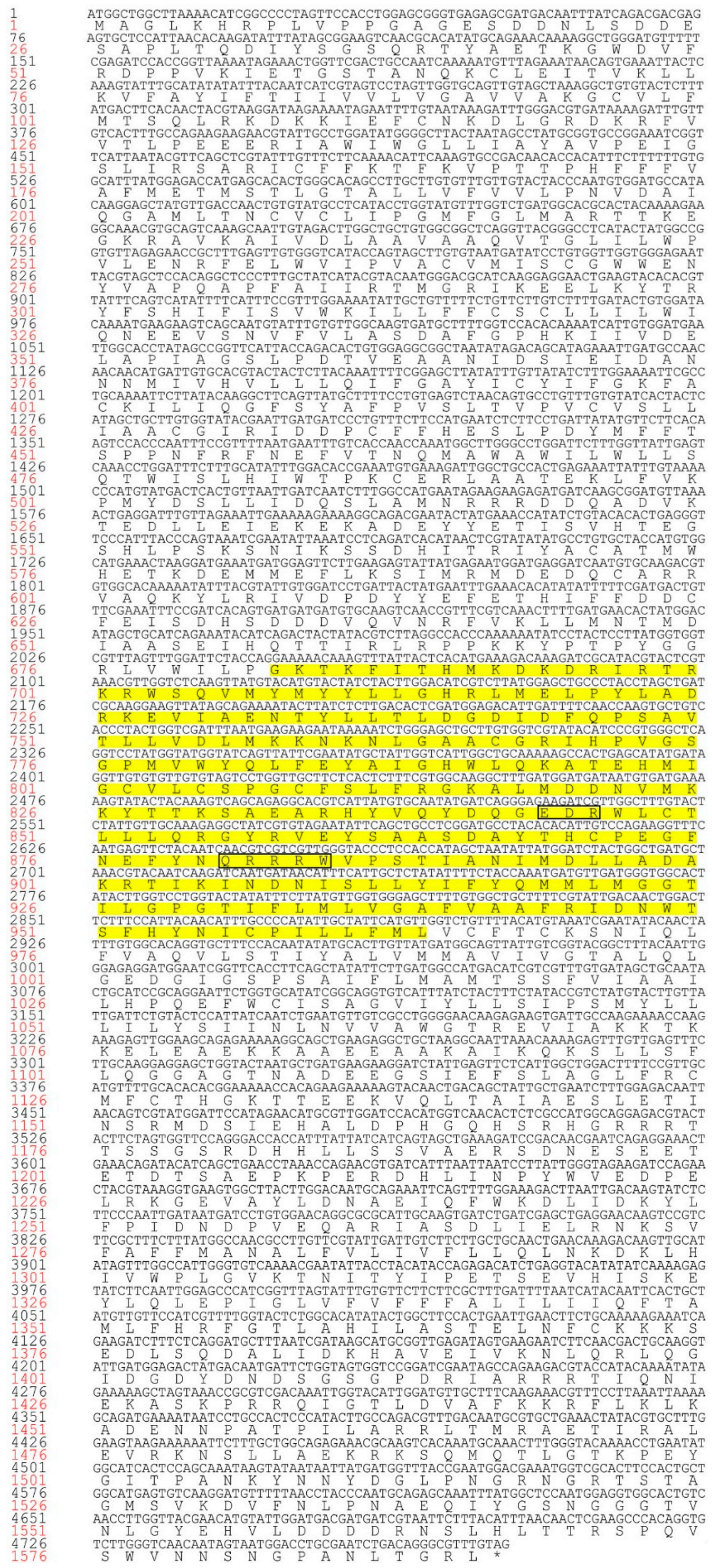

Figure S1 Nucleotide sequences for *L. trifolii* CHS2 cDNA and its predicted amino acid sequence. Nucleotide numbering starts with the adenine in the first methionine codon of the putative open reading frame. The highly conserved region, chitin_synth_2 domain, is highlighted. The asterisk indicates the translational termination codon. The EDR and QRRRW motifs are boxed.
